# Mitophagy in ischemic heart disease: molecular mechanisms and clinical management

**DOI:** 10.1038/s41419-024-07303-3

**Published:** 2024-12-30

**Authors:** Shujuan Xu, Zihan Wang, Fan Guo, Yehao Zhang, Han Peng, Huiyu Zhang, Zixin Liu, Ce Cao, Gaojie Xin, Yuan Yuan Chen, Jianhua Fu

**Affiliations:** 1 Xiyuan Hospital of China Academy of Chinese Medical Sciences, Beijing, 100091 China; 2 Department of Oral Implantology, School and Hospital of Stomatology, China Medical University, Liaoning Provincial Key Laboratory of Oral Diseases, Shenyang, 110122 China

**Keywords:** Autophagy, Diseases

## Abstract

The influence of the mitochondrial control system on ischemic heart disease has become a major focus of current research. Mitophagy, as a very crucial part of the mitochondrial control system, plays a special role in ischemic heart disease, unlike mitochondrial dynamics. The published reviews have not explored in detail the unique function of mitophagy in ischemic heart disease, therefore, the aim of this paper is to summarize how mitophagy regulates the progression of ischemic heart disease. We conclude that mitophagy affects ischemic heart disease by promoting cardiomyocyte hypertrophy and fibrosis, the progression of oxidative stress, the development of inflammation, and cardiomyocyte death, and that the specific mechanisms of mitophagy are worthy of further investigation.

## Facts

1. Mitophagy, as a crucial part of the mitochondrial control system, plays a special role in ischemic heart disease, unlike mitochondrial dynamics.
2. Mitophagy mainly affects ischemic heart disease by promoting myocardial cell hypertrophy and fibrosis, promoting oxidative stress progression, promoting inflammation, and promoting myocardial cell death.
3. Compared to traditional Western medicine, traditional Chinese medicine can indeed effectively reduce kidney toxicity. Focusing on how to combine traditional Chinese medicine and Western medicine to treat ischemic cardiomyopathy on the existing basis, we believe that the combination of the two can effectively improve the protection of myocardial cells.

## Open question

1. What is the proportion of mitochondrial autophagy in ischemic heart disease? This is something that needs to be clearly confirmed in the future.
2. Does mitochondrial autophagy still play a double-edged sword role in ischemic heart disease?
3. Most of the achievements in mitophagy affecting ischemic cardiomyopathy currently have their own characteristics and are not yet sufficient to be generalized to all patients with ischemic heart disease.
4. The clinical translation of mitophagy in ischemic heart disease still needs to be further strengthened.

## Introduction

### Mitophagy

#### Introduction to mitophagy

The mechanism of autophagy was identified *via* fluorescence microscopy in 2004 [1]. Autophagy, defined as “the process by which a cell engulfs itself,” is categorized into micro-autophagy, macro-autophagy, and chaperone-mediated autophagy. Depending on the size and specificity of the targeted cellular components, autophagy can be either non-selective or selective [2]. The mitochondrion, known as the powerhouse of the cell, is a dual-membrane, dynamic organelle essential for cellular metabolism [3, 4]. Damaged mitochondria or those under certain developmental conditions are eliminated through a selective form of autophagy called mitophagy [5]. Initially described in yeast, mitophagy was shown to be obstructed by the presence of a mutant Uth1p in the outer mitochondrial membrane (OMM) during starvation [6]. Similar findings were later observed in cultured starved hepatocytes, where oxidative damage prompted the removal of damaged mitochondria to maintain mitochondrial integrity for cellular homeostasis [7]. However, mitochondrial phagocytosis not only addresses dysfunctional mitochondria but also reduces overall mitochondrial mass under specific stresses such as hypoxia, nutrient deprivation, and exposure to mitochondrial uncouplers4. This process decreases the mitochondrial pool by mutations in the OMM, thereby preventing the production of reactive oxygen species (ROS), conserving vital resources like oxygen, and promoting cell survival under stress conditions [8].

#### Mechanism of mitophagy

##### BNIP3 and NIX

BNIP3 and BNIP3-like protein (BNIP3L)/Nip3-like protein X (NIX), members of the BCL2 family of BH3-only domain proteins, are localized to the OMM and function as pro-apoptotic proteins. They share 56% homology and possess an N-terminal amino acid sequence capable of binding to LC3 [9]. BNIP3 is essential for efficient mitochondrial adaptation to hypoxia [10]. The N-terminal domain of BNIP3, a mitochondrial protein, is up-regulated in response to hypoxia and anchored to the OMM through its C-terminal transmembrane (TM) domain, exposing the N-terminal domain to the cytoplasm [11]. Normally expressed as an inactive monomer in the cytoplasm, BNIP3 forms a stable homodimer *via* its C-terminal TM domain under stress, integrating into the OMM [12]. The N-terminal region of BNIP3 contains an LC3-interacting region (LIR) motif, and mutations in this region disrupt its interaction with LC3, leading to impaired mitophagy.

NIX, another BNIP3 protein in the BCL2 family, facilitates selective mitochondrial degradation during reticulocyte maturation [13, 14]. Due to the high sequence similarity between BNIP3 and NIX, BNIP3 expression can restore mitochondrial clearance in the absence of NIX [15]. Similar to BNIP3, NIX dimerization, regulated by phosphorylation of its C-terminal region, is essential for the effective functioning of the mitophagy machinery [16]. Despite their similarities, NIX/BNIP3L does not fully compensate for BNIP3 depletion. NIX/BNIP3L has been shown to be involved in the PINK1-parkin pathway, being ubiquitinated by parkin and recruiting the mitochondrial phagocytosis receptor, NBR1, for mitochondrial degradation [17]. These results suggest potential crosstalk between different mitochondrial phagocytosis pathways.

##### FUNDC1

FFUNDC1, a mitochondrial outer membrane protein, induces mitophagy in mammalian cells under low oxygen conditions [18]. It features a typical LIR motif near the N-terminal region and three transmembrane (TM) domains [18]. The mitophagic activity of FUNDC1 is regulated by phosphorylation and ubiquitination. Under normoxic conditions, SRC (sarcoma gene) receptor kinase and casein kinase II (CK2) phosphorylate FUNDC1 at Tyr18 and Ser13, respectively, inhibiting mitophagy. Conversely, under hypoxic conditions, phosphoglycerate mutase 5 (PGAM5) and ULK1 catalyze the dephosphorylation and phosphorylation of Ser13 and Ser17, respectively, thereby inducing mitophagy [19]. Additionally, membrane-associated RING finger protein 5 (MARCH5) catalyzes the phosphorylation of FUNDC1 at Tyr18 and Ser13, inhibiting mitophagy, while promoting its ubiquitination and degradation at Lys119 under prolonged hypoxia, allowing residual FUNDC1 to mediate mitophagy [20].

##### BCL2L13

BcL2L13, also known as BCL-Rambo, is an OMM protein and member of the Bcl-2 family, comprising four BH domains (BH1-4), the BHNo region, and a transmembrane domain [21]. Bcl-rambo, the mammalian homolog of ATG32, binds to LC3 through the WxxI motif, mediating mitophagy independently of ubiquitination [21]. While no direct homologs of Atg32 have been identified in mammalian cells, yeast studies suggest that BCL2L13 can induce mitophagy in Atg32-deficient cells, implying that BCL2L13 might functionally complement Atg32 in mammals [21].

##### FKBP8

FKBP8, also known as FKBP38, is a member of the FKBP family, localized on the mitochondrial outer membrane [22]. It contains a typical LIR motif near the N-terminal end and a TM domain at the C-terminal end. FKBP8 mediates mitophagy by binding to LC3A through its LIR motif, independent of the PINK1/Parkin pathway, which is essential for its mitophagic activity [22]. Notably, FKBP8 translocates from damaged, acidified mitochondria to the endoplasmic reticulum to avoid degradation, indicating that this translocation relies on the weakly basic sequence at the C-terminal end, thereby inhibiting apoptosis during autophagy [23]. Given the multifunctional complexity of FKBP8, further studies are necessary to determine its direct involvement in mitophagy.

##### Parkinson protein 2-dependent pathway

PTEN-induced putative kinase 1 (PINK1/PARK6) and Parkin (PARK2), initially identified as key proteins associated with Parkinson’s disease, have been recognized for their pivotal roles in mitophagy [24]. Parkin functions as an E3 ubiquitin ligase [25]. Research has demonstrated that PINK1 is an upstream regulator of Parkin, with both proteins synergistically mediating the polyubiquitination of structural or functional proteins on the surface of damaged mitochondria, playing a pivotal role in the autophagic degradation of depolarized mitochondria [26]. PINK1, synthesized in the cytoplasm, is present at low levels in healthy mitochondria. However, when mitochondria are depolarized or damaged, PINK1 accumulates in large quantities in the OMM, translocating Parkin from the cytoplasm to the OMM by up-regulating its autophosphorylation and phosphorylating Parkin (ubiquitin, Ub) *via* its C-terminal kinase region [27]. PINK1 activates through autophosphorylation and phosphorylates serine residues on adjacent ubiquitin molecules. These phosphorylated ubiquitin molecules bind to and recruit Parkin, which is then phosphorylated and activated by PINK1. Activated Parkin polyubiquitinates various mitochondrial protein substrates [28]. In the presence of LC3 junction proteins, autophagosomes target mitochondria, inducing mitophagy. While p62 acts as an adapter between ubiquitinated proteins and autophagy, its expression level as a reflection of autophagy activation remains controversial.

#### Mitophagy and mitochondrial dynamics

Mitochondria are highly dynamic organelles undergoing constant motility, fission, and fusion in response to environmental stimuli. Before selective elimination, damaged mitochondria are asymmetrically divided into healthy and damaged organelles by fission proteins. These fission proteins include Drp1, FIS1, and MFF [29]. Damaged organelles are degraded through mitophagy, while healthy ones can fuse with other mitochondria to mix contents and maintain the genetic integrity of mitochondrial fission proteins. Severely damaged mitochondria are replaced by new mitochondria produced through mitochondrial biogenesis [30]. Mitochondrial fusion involves the merging of outer and inner membranes, facilitated by mitofusins (Mfn1, Mfn2) and OPA1. This dynamic process is known as mitochondrial dynamics [31].

**Fig. 1:**
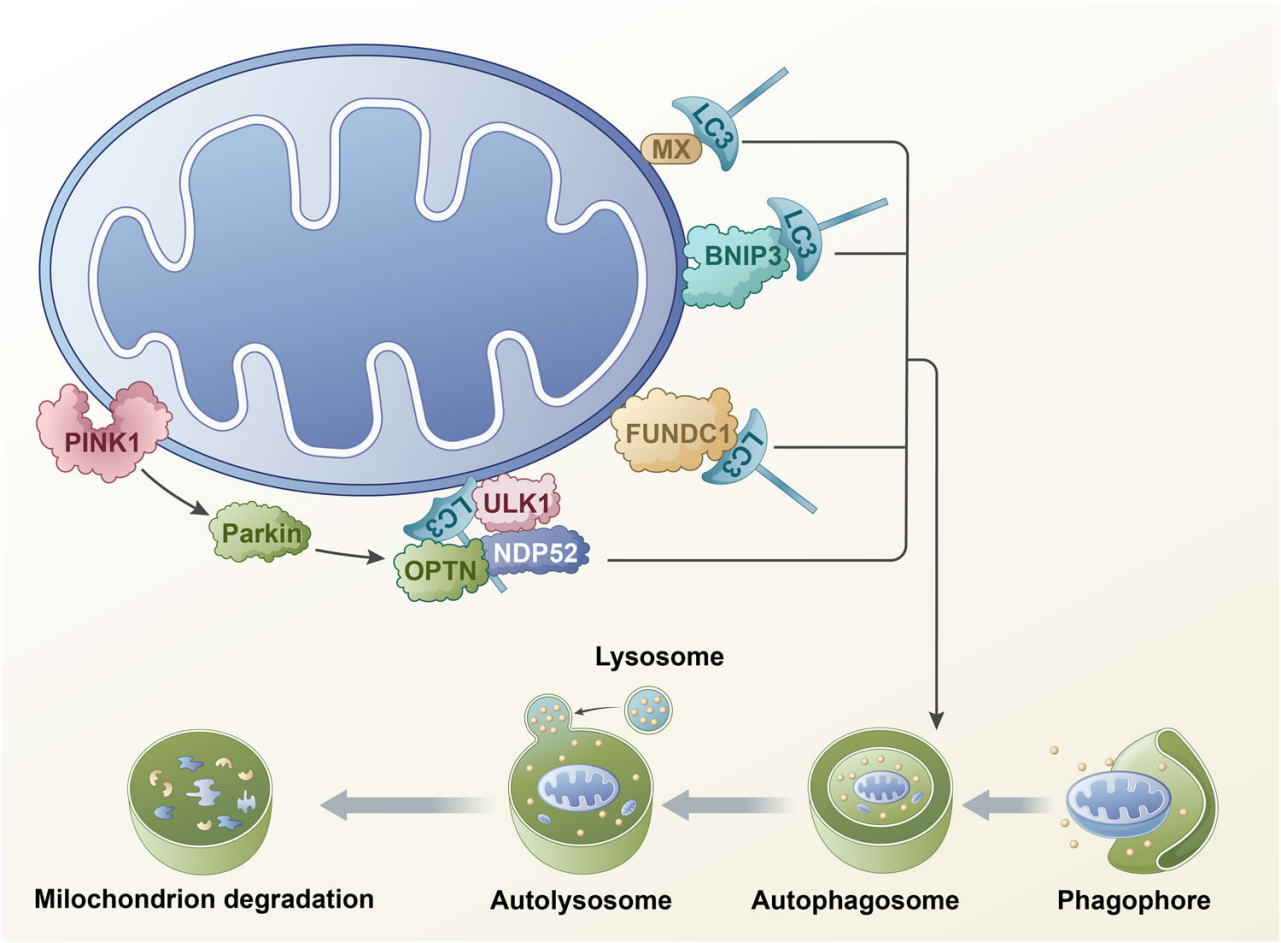
The mechanism of mitophagy. Mitophagy can be divided into approximately four key steps: 1. In the early stage of mitochondrial damage, permeability transition occurs, leading to mitochondrial depolarization, loss of membrane potential, and induction of mitochondrial autocorrelation protein activation. 2. In the early stage, autophagosomes wrap around damaged mitochondria, forming mitophagy. 3. In the middle stage, mitochondrial autophagosomes fuse with lysosomes to form mature mitochondrial autophagosomes. 4. Lysosomal acidic hydrolytic enzymes flow into autophagosomes to degrade mitochondria, allowing nutrients to be recycled and reused. The molecular mechanism of mitophagy can be mainly divided into ubiquitin dependent pathway and non ubiquitin dependent pathway. The key proteins in the ubiquitin dependent pathway are PINK1 and Parkin. In addition, besides the PINK1 Parkin pathway, there is also a non Parkin dependent ubiquitin dependent pathway. That is to say, PINK1 can also recruit self receptor proteins (such as NIX, BNIP3, and FUNDC1) directly to mitochondria through ubiquitin phosphorylation, and the receptor proteins recruit LC3, which enables the self to engulf mitochondria. Non ubiquitin dependent mitophagy is dominated by mitophagy receptors, which differs significantly from the ubiquitin dependent pathway.

Mitochondrial dynamics and mitophagy are essential processes that maintain mitochondrial homeostasis and normal physiological functions (Fig. 1). Extensive evidence indicates that inhibiting mitochondrial fission or enhancing mitochondrial fusion reduces mitophagy [32]. Numerous studies have established that mitochondrial fission is a prerequisite for mitophagy [33]. Downregulation of Drp1 through Ad-shDrp1 transfection significantly decreased Lc3 protein levels in cardiomyocytes [34]. LC3 protein is a direct indicator for assessing the degree of autophagy. Consequently, reduced autophagy due to Lc3 downregulation leads to the accumulation of dysfunctional mitochondria in cardiomyocytes [35]. BNIP3, another mitophagy-associated protein, induces autophagy [35]. Overexpression of BNIP3 increases Drp1-mediated mitochondrial fission [36]. FUNDC1 regulates mitochondrial fission by interacting with OPA1 [37]. Overexpression of FUNDC1 promotes mitochondrial fission and mitophagy, while its knockdown induces mitochondrial fusion. The normal mitochondrial dynamic network balances mitochondrial fission and fusion. Other studies have shown that regulating mitochondrial fusion similarly affects mitophagy as mitochondrial fission [37, 38]. For instance, OPA1 knockdown-induced reduction in fusion also facilitates the elimination of dysfunctional mitochondria [39]. However, conditional knockdown of Mfn1/2 in mouse cardiomyocytes results in increased mitochondrial dysfunction and cardiac hypertrophy due to impaired mitophagy [40]. This study demonstrated that mitochondrial fusion is a key component of mitochondrial dynamics, and thus, mitochondrial dynamics is integral to mitophagy. Notably, mitochondrial dynamics can also regulate mitophagy correspondingly.

#### Mitophagy and mitochondrial biogenesis

Mitophagy and mitochondrial biogenesis are interrelated processes with opposing roles in regulating mitochondrial number and content [41]. Excessive mitophagy or elevated mitochondrial biogenesis disrupts the delicate balance of mitochondrial homeostasis, ultimately leading to cell death or necrosis in the case of mitophagy [42]. Mitophagy is the predominant cause of mitochondrial turnover.

AMPK and CaMK are compounds that regulate mitophagy and directly activate PGC-1 to enhance mitochondrial biogenesis [43]. Additionally, AMPK-induced NAD+ stimulation increases PGC-1 levels [44]. These proteins influence PGC-1 through post-translational modifications. NAD^+^ activates SIRT1, which deacetylates PGC-1 in response to mitochondrial metabolism [45]. As a bridge between mitophagy and mitochondrial biogenesis, AMPK also phosphorylates ULK1/2 to initiate autophagic vesicle formation [46]. Upon Ca^2+^ increase, CaMK enzymes participate in mitophagy by stimulating AMPK [47]. SIRT1 overexpression stimulates autophagosome formation, with lysosomes acting as the ultimate “incinerators” that degrade mitochondria during clearance [48]. Additionally, there is a positive feedback loop between TFEB and PGC-1 interaction through the CLEAR (Coordination of Lysosomal Expression and Regulation) motif, which maintains mitochondrial number and content stability [49]. The CLEAR motif is present not only in human or mouse mitochondrial genes but also in other autophagy-related genes. Studies have shown that TFEB regulates mitochondrial biogenesis through PGC-1 and serves as a therapeutic target for cardiac disease [50]. Moreover, PGC-1 activator ZLN005 mitigates renal injury by accelerating mitochondrial clearance in mice with cisplatin-induced AKI, increasing co-localization between LC3 and damaged mitochondria [51]. PGC-1 is also known for its protective effects in myocardial ischemia-reperfusion injury and heart failure, although cardiac PGC-1 agonist drugs are not well established. Mice deficient in cardiac PGC-1 are reported to be more susceptible to heart failure than those deficient in systemic PGC-1, though the underlying reason remains unclear [51]. Future breakthroughs may be achieved by considering the role of mitophagy in this context.

#### mitophagy and the granule permeability transition pore (mPTP)

Both the mitochondrial permeability transition pore (mPTP) and mitophagy play important roles in the regulation of cellular metabolism and death, particularly in ischemic diseases such as myocardial infarction and stroke. These mechanisms are critical for cell fate and can influence the course of disease by modulating mitochondrial function and cell death.

The opening of mPTP, a multiprotein complex on the inner mitochondrial membrane, leads to a loss of transmembrane potential in and out of the mitochondria, which in turn causes mitochondrial swelling and rupture of the outer membrane [52]. The opening of mPTP is often regarded as a hallmark of cell death, especially when the cell is subjected to severe injury or stress. mPTP opening can be triggered by a variety of factors, including calcium ion overload, oxidative stress and decreased ATP levels [53]. Certain proteins such as cyclophilin D (Cyclophilin D) regulate the opening of mPTP and are potential drug targets [54]. Following cardiac or cerebral ischemia, with rapid depletion of oxygen and nutrients, there are elevated levels of intracellular Ca^2+^ and increased oxidative stress, changes that promote the opening of mPTP [55]. The sustained opening of mPTP leads to loss of mitochondrial function, which ultimately triggers apoptosis or necrosis and exacerbates tissue damage.

Under ischemic conditions, the opening of mPTP may act as a signal for mitochondrial quality control, triggering mitophagy [56]. Mitochondrial swelling and loss of potential due to open mPTP is one of the signals for mitophagy to recognize damaged mitochondria [57]. Molecules released from mitochondria after the opening of mPTP, such as cytochrome c, may further promote autophagosome formation and accelerate the mitochondrial clearance. mitophagy reduces cellular damage by removing mitochondria that are open to mPTP and is a protective mechanism, especially in the context of postischemic reperfusion [57].

Therefore, by targeting mPTP and optimizing the regulation of mitophagy, new therapeutic strategies may be developed to mitigate the damage caused by these conditions.

#### mitophagy in endothelial and cardiomyocytes

Mitophagy is present in both endothelial and cardiomyocytes under physiological and pathological conditions, but the two cell types exhibit differences in certain key aspects. Key to these differences and similarities are their respective physiological functions, the microenvironment in which they are found, and the way in which they respond in response to stress.

In terms of physiological function, endothelial cells are primarily responsible for blood vessel formation and maintenance as well as mediating blood flow and vascular permeability. Therefore, mitophagy in endothelial cells may be more associated with cell migration, proliferation, and angiogenic processes [58]. Cardiomyocytes, on the other hand, are primarily responsible for the contractile function of the heart, and mitophagy in cardiomyocytes is mainly used to maintain the highly demanded energy production and to ensure the effectiveness of the heart’s pumping functions [59].

Under pathological conditions, abnormal activation or inhibition of mitophagy in cardiac myocytes in heart disease such as myocardial infarction may have a profound effect on cardiac function [60]. In contrast, mitophagy in endothelial cells may be more likely to affect vascular lesions such as atherosclerosis or vascular complications associated with diabetes mellitus [61].

More critically, the dependence of cardiomyocytes on mitochondria is more pronounced in ischemia and reperfusion injury due to the extremely high demand for energy supply in the heart [58]. As a result, mitophagy in cardiomyocytes may be more intense and frequent. Whereas endothelial cells may be more involved in vascular reconstruction and repair under ischemic conditions, the regulation of mitophagy may be more focused on modulating cellular barrier function and vascular responses [62].

In conclusion, endothelial cells and cardiomyocytes show some commonalities in mitophagy, especially in maintaining mitochondrial function and responding to cellular stress. However, due to their different roles in the cardiovascular system, there are significant differences in the regulation and functional expression of mitophagy between the two, and these differences reflect their unique responses and adaptive mechanisms to specific pathological states.

#### Overview of mitophagy and heart disease

The cytoplasm of normal cardiomyocytes is rich in round or oval mitochondria that are densely and neatly arranged and fill the entire lumen In general, mitochondrial swelling or vesicular degeneration is usually very rare. Once cardiomyocytes are subjected to intolerable hypoxic stress or other factors, irreversible mitochondrial damage will result, which in turn leads to mitochondrial swelling, deformation, and rupture, thus directly affecting mitochondrial energy metabolism. Since cardiomyocytes are non-renewable cells with a high energy demand, cardiomyocytes are damaged when mitochondrial energy metabolism is dysregulated.

Current research evidence supports that myocardial injury and vascular endothelial dysfunction in ischemic cardiomyopathy are closely related to mitochondrial plasmonic dysregulation, which is involved in the regulation of cardiomyocyte physiopathological functions [63]. It has also been demonstrated that mitophagy is capable of interfering with cardiomyocyte survival by affecting DRP1 [64]. Similarly, mitophagy is capable of interfering with the Hippo pathway to activate cardiomyocyte injury [65]. In response to stress-induced injury, the mitochondrial quality control system is activated, encompassing mitochondrial dynamics, mitophagy, and mitochondrial biogenesis, to restore homeostasis by inducing fission, degradation, or regeneration [66]. Aberrations in this system, such as excessive mitochondrial fission, defective mitophagy, and delayed biogenesis, are associated with additional cardiomyocyte injury and thus represent potential targets for heart disease therapy [67]. Both excessive and insufficient mitophagy contribute to cellular damage, while moderate mitophagy is essential for maintaining mitochondrial health [33].

Investigating the mechanisms of mitophagy in heart diseases is essential. The prevailing view is that mitophagy influences heart failure, myocardial infarction, ischemia-reperfusion injury, cardiomyopathy, atherosclerosis, metabolic cardiovascular diseases, cardiovascular aging, and other conditions [68, 69]. Dysregulated mitophagy affects the pathogenesis of cardiovascular diseases, leading to mitochondrial damage, reactive oxygen species (ROS) production, and ultimately the onset or progression of various cardiovascular disease subtypes.

This review focuses on exploring the mechanisms of mitophagy in ischemic heart disease, summarizing recent studies on the protective role of mitophagy, and highlighting promising therapeutic targets.

## II. Mechanism of action of mitophagy in promoting the progression of ischemic heart disease

### Mitophagy promotes cardiomyocyte hypertrophy and fibrosis

Myocardial fibrosis is a pathological alteration in the heart’s structure and morphology caused by various myocardial injuries. Studies have shown that combined treatment with metformin (MET) and ginseng dingzhi tang (GN) in hyperglycemic cardiomyocytes can indirectly enhance mitophagy, thereby reducing myocardial injury^58^. Mitochondrial fission induced by Drp1 has been demonstrated to cause mitophagy dysfunction, leading to cardiomyocyte hypertrophy [70]. Deficiency in Mfn1 or Mfn2 expression disrupts mitochondrial structure, promoting cardiomyocyte hypertrophy and heart failure, and significantly inhibiting the expression of mitochondrial biogenesis-related proteins (PGC-1α and Nrf-1) [71].

Similarly, levels of mitophagy proteins (PINK1 and FUNDC1) are significantly downregulated during cardiac hypertrophy. Conditional knockdown of Drp1 in cardiomyocytes inhibits mitochondrial fission and induces mitochondrial enlargement [72]. In mice subjected to transverse aortic constriction (TAC), a model of pressure overload-induced heart failure, cardiomyocytes exhibit severe hypertrophy and mitophagy activation.

However, at later stages, mitophagy is inhibited, leading to severe mitochondrial dysfunction and heart failure. Injection of the autophagy inducer Tat-Beclin-1 increases mitophagy, reducing mitochondrial dysfunction, cardiomyocyte hypertrophy, and heart failure induced by pressure overload [73]. These results suggest that abnormal mitophagy may contribute to cardiomyocyte hypertrophy.

Contrastingly, mitophagy plays a partial role in myocardial fibrosis. Excessive mitochondrial fission is believed to enhance fibroblast proliferation and collagen production, likely because mitochondrial fission acts downstream of TGF-β1 [74]. Inhibition of mitophagy by Mdivi-1 significantly attenuates fibroblast proliferation, collagen production, and fibrosis in the border zone of myocardial infarction [34]. Unlike mitochondrial fission, pharmacological activation of mitophagy is associated with angiotensin II and atrial natriuretic peptide levels, reducing cardiomyocyte apoptosis and myocardial fibrosis in a model of pressure overload-induced chronic cardiac insufficiency [75].

### Mitophagy promotes oxidative stress progression

**Fig. 2:**
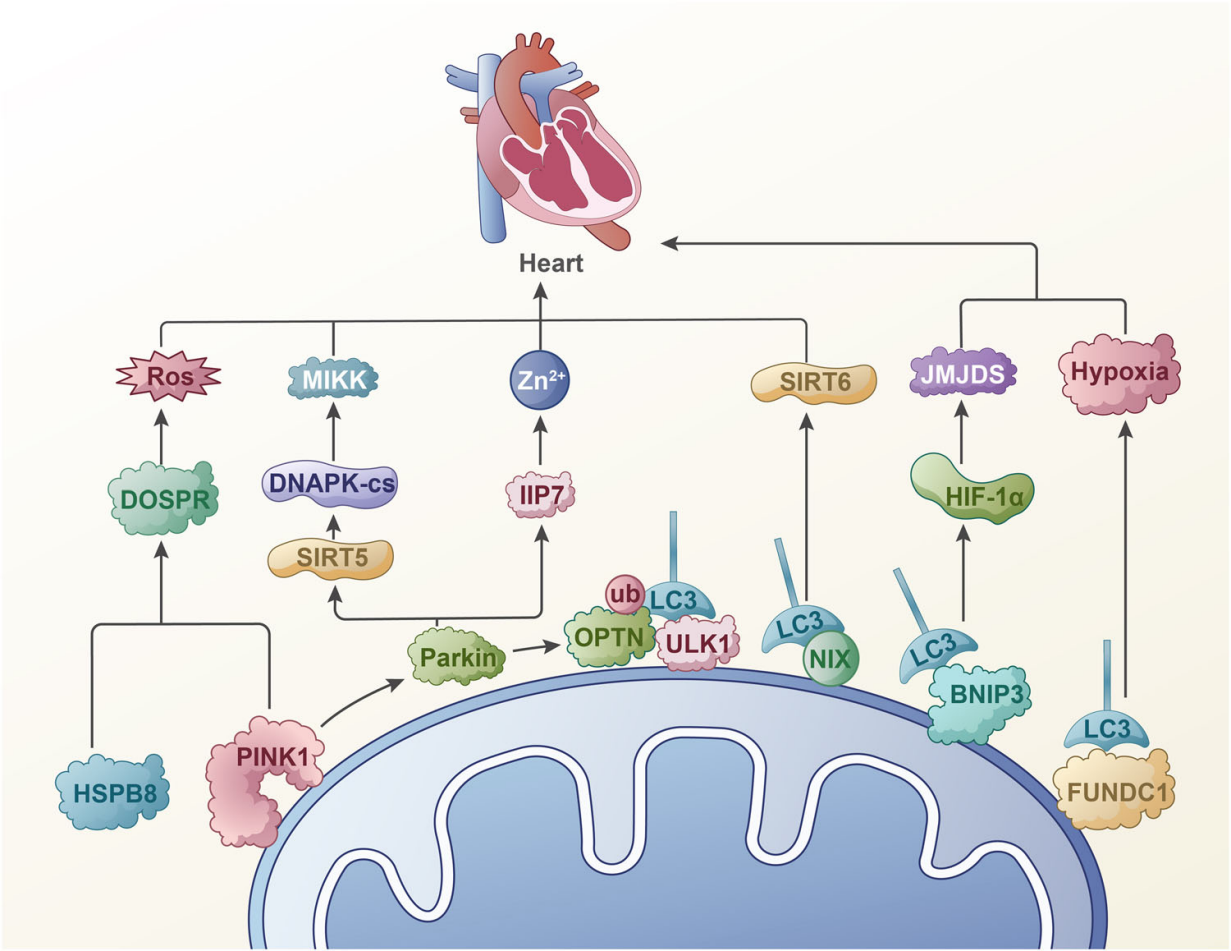
Mitophagy promotes oxidative stress in ischemic cardiomyopathy. The inflammatory signals in myocardial cells usually begin with the accumulation of myocardial cell mitochondria caused by stress response. Research has shown that YQHX can alleviate hypoxia induced damage by targeting mitophagy. On the other hand, quercetin alleviates mitochondrial oxidative stress through DNA-PKcs-SIRT5. DUSP12 can inhibit cell apoptosis caused by hypoxia through HSPB8. Inhibition of Parkin mediated mitophagy leads to excessive accumulation of mitochondrial ROS, which in turn promotes the progression of progressive myocardial injury and heart failure. Under low oxygen conditions, mitophagy regulates myocardial ischemia-reperfusion injury through the HIF/BNIP3 pathway. Similarly, studies have shown that JMJD5 can alleviate myocardial cell damage by regulating the HIF-BNIP3 pathway. Other studies have shown that exosomes rich in Sirt6 inhibit cell pyroptosis in AIM2 and enhance mitophagy through the p62 and Beclin-1 pathways, thereby improving myocardial cell damage. The knockdown of ZIP7 has also been confirmed to reduce mitochondrial ROS generation and myocardial infarction by increasing Zn^2+^in mitochondria, leading to mitochondrial depolarization, as well as the accumulation of PINK1 and Parkin in mitochondria.

Inflammatory signaling in cardiomyocytes typically initiates with an early stress response to myocardial injury, leading to the accumulation of mitochondrial ROS and subsequent impairment of mitochondrial oxidative phosphorylation [76]. Elevated myocardial ROS levels are observed in ischemia-reperfusion injury, acute myocardial infarction, and heart failure in animal models of cardiac disorders [77]. In vitro studies also demonstrate that hypoxia, inflammatory, and hyperglycemic conditions further elevate ROS levels, indicating that ROS is a critical factor in disrupting mitochondrial homeostasis [78]. As by-products of normal aerobic metabolism, ROS can activate various mitochondrial signaling pathways, such as Nrf2/HIF-1/NF-κB, even at low concentrations [79]. Thus, studying the association between mitophagy and ROS in ischemic cardiomyopathy is essential (Fig. 2).

Recent research indicates that Yiqihuoxue (YQHX) alleviates hypoxia-induced injury and attenuates oxidative stress by targeting mitophagy [59]. This highlights the connection between mitophagy and hypoxic injury in cardiovascular disease. Administration of moderate mitochondria-targeted catalase has been shown to inhibit mitochondrial ROS accumulation, membrane depolarization, and structural and functional damage. However, high doses of catalase, while inhibiting mitochondrial ROS expression, did not improve mitochondrial function or inhibit cardiomyocyte apoptosis [80]. This suggests that maintaining mitochondrial ROS at critical levels is necessary for mitochondrial homeostasis. Additionally, quercetin has been shown to mitigate mitochondrial oxidative stress-induced cardiomyocyte injury and inhibit necrotic apoptosis in cardiomyocytes post-ischemia/reperfusion (I/R) *via* the DNA-PKcs-SIRT5 pathway [81]. Similarly, DUSP12 ameliorates myocardial I/R injury by inhibiting hypoxia-induced apoptosis *via* HSPB8, thereby increasing mitophagy [82]. Melatonin inhibits mitophagy, reduces apoptosis and oxidative stress, and restores mitochondrial function [83]. Interestingly, melatonin-induced cardioprotection in hypoxia-induced ischemic cardiomyopathy is independent of changes in the PINK1/Parkin pathway [84].

Further exploration of the pathways through which ROS influences mitophagy to alter the progression of ischemic cardiomyopathy has shown that inhibition of Parkin-mediated mitophagy leads to excessive mitochondrial ROS accumulation, promoting myocardial injury and heart failure [85]. In a hypoxia-mediated environment, mitophagy is linked with the onset of myocardial ischemia-reperfusion injury *via* the HIF-1/BNIP3 pathway [86]. Studies have demonstrated that JMJD5 can attenuate oxygen-glucose deprivation and reperfusion-induced injury in cardiomyocytes by modulating the HIF1α-BNIP3 pathway [87]. Hypoxia induces extensive mitochondrial degradation in platelets in a FUNDC1-dependent manner, with hypoxic mitophagy in platelets protecting the heart from worsening ischemia-reperfusion injury [88]. This underscores the role of mitophagy in mitochondrial quality control and platelet activation, suggesting that manipulating mitophagy through hypoxia or pharmacological approaches could be a novel cardioprotective strategy. Furthermore, Sirt6-enriched exosomes have been shown to inhibit cellular focal death in AIM2 and enhance mitophagy *via* the p62 and Beclin-1 pathways, thereby ameliorating myocardial ischemia-reperfusion injury [89].

As a secondary calcium reservoir within cardiomyocytes, mitochondria can buffer Ca^2+^ in response to fluctuations in cytosolic calcium levels. Excessive mitochondrial calcium accumulation disrupts oxidative phosphorylation and ATP synthesis, impairing cardiomyocyte contraction and relaxation [90]. Increased mitochondrial fission triggers oxidative stress in ischemic cardiomyocytes. Mechanistically, mtDNA is unevenly distributed in daughter mitochondria during fission, potentially reducing transcription of respiratory complex-related proteins and leading to excessive mtROS production [91]. mtROS-mediated oxidative damage results in cysteine residues within the mitochondrial calcium uniporter (MCU) becoming cysteinylated with S-glutathione, assembling the MCU channel into persistently active higher-order complexes, and promoting sustained mitochondrial calcium uptake [92]. Additionally, sarcoplasmic/endoplasmic reticulum Ca^2+^-ATPase (SERCA), which pumps calcium from the cytoplasm back into the sarcoplasmic/endoplasmic reticulum, is susceptible to oxidation at cysteine-674, resulting in intracellular calcium overload [93]. Mitophagy-mediated mitochondrial removal significantly reduces mtROS production. Although there is a causal relationship between oxidative stress and intracellular/mitochondrial calcium overload, evidence supporting a regulatory role of mitophagy in calcium handling is lacking. Some studies suggest moderate mitochondrial calcium accumulation promotes mitochondrial biogenesis, yet the impact of biogenesis on calcium handling warrants further investigation. Mitochondrial biogenesis-associated proteins, particularly Nrf-1/2, act as transcription factors enhancing the expression of mitochondrial antioxidant genes (including manganese superoxide dismutase, catalase, peroxiredoxins 3 and 5, uncoupling protein 2, thioredoxin 2, and thioredoxin reductase), highlighting the antioxidant properties of mitochondrial biogenesis [94].

Interestingly, like Ca^2+^, Zn^2+^ plays a vital role in regulating mitochondrial oxidative stress. Knockdown of ZIP7 reduces mitochondrial ROS generation and myocardial infarction by increasing Zn^2+^ in mitochondria, leading to mitochondrial depolarization and the accumulation of PINK1 and Parkin [95]. Chronic overactivation of HIF1, increased cytoplasmic and mitochondrial calcium overload, and other dysregulations of mitophagy exacerbate ischemia-reperfusion injury. Furthermore, iron metabolism plays a key role in mitophagy-mediated myocardial ischemia [96].

**Fig. 3:**
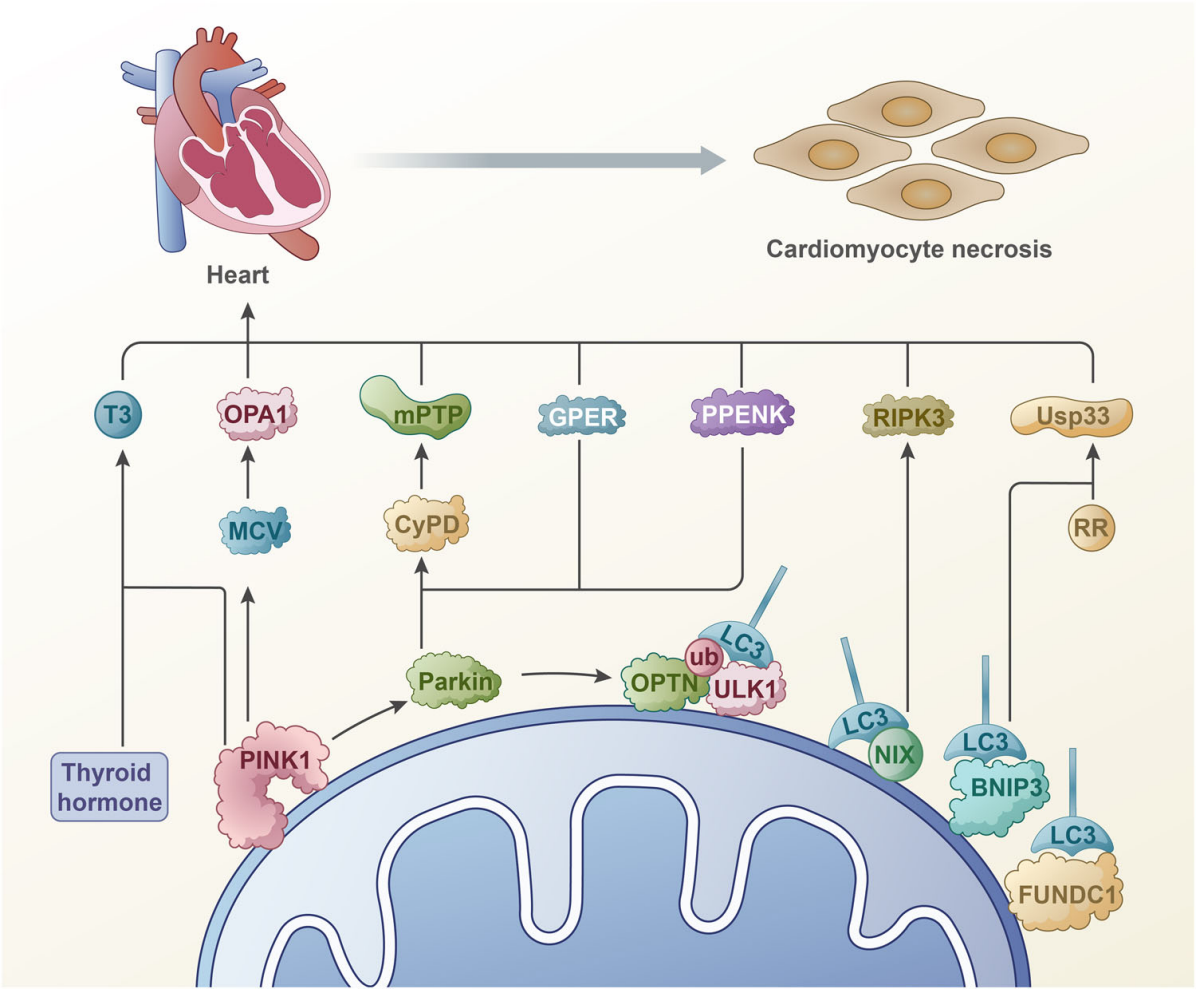
Mitophagy promotes myocardial cell death. The research results indicate that TBC1D15 plays a key role in myocardial injury through mitophagy regulated by Fis1/RAB7. Parkin inhibits mPTP opening and myocardial cell necrosis by catalyzing the ubiquitination of CypD. Upregulation of MCU helps to inhibit calpain/OPA-1 mediated mitophagy and suppress excessive cell apoptosis through OPA1. Thyroid hormones can provide cardiac protection by enhancing PINK1 dependent mitophagy. MiR-494-3p can target and negatively regulate PGC1- α mediated mitophagy in cardiomyocytes, inhibiting cardiomyocyte apoptosis. Rich hydrogen saline alleviates inflammation and cell apoptosis in myocardial I/R injury through PINK1/Parkin mediated mitophagy. RR can inhibit cell apoptosis by inhibiting USP33 to promote mitophagy. Similarly, PPENK can promote mitophagy and reduce myocardial ischemia-reperfusion injury through the PINK1 Parkin pathway. Drp1 induced mitophagy disruption has tolerance to hypoxia induced damage. RIPK3 inhibits AMPK to prevent PINK1-PRKN induced mitophagy, thereby promoting myocardial cell necrosis. Ischemia reperfusion can trigger upregulation of RIPK3, promote phosphorylation of FUNDC1, and induce cell apoptosis.

### Mitophagy promotes myocardial cell death (Fig. 3)

The findings indicate that TBC1D15 is instrumental in acute myocardial infarction-induced cardiac abnormalities through Fis1/RAB7-regulated mitochondrial-lysosomal contact, subsequently activating lysosome-dependent mitochondrial autophagic fluxes [97]. Parkin mediates mitophagy and inhibits necrosis in response to oxidative stress by catalyzing the ubiquitination of cyclophilin D (CypD) within the necroptosis cascade, thereby preventing mPTP opening and improving cardiac function [98]. Upregulation of the mitochondrial calcium uniporter (MCU) contributes to calpain/OPA-1-mediated inhibition of mitophagy, suppressing excessive apoptosis through OPA1 [99]. Thyroid hormone treatment enhances PINK1-dependent mitophagy, offering cardiac protection and reducing cell death [100]. Additionally, Parkin-mediated mitophagy mitigates oxidative stress, providing cardioprotection against I/R injury by reducing cell death [100]. miR-494-3p targets and negatively regulates PGC1-α, alleviating myocardial ischemia-reperfusion injury and inhibiting cardiomyocyte apoptosis through modulating mitophagy [101]. Hydrogen-rich saline mitigates myocardial I/R injury *via* PINK1/Parkin-mediated mitophagy by reducing inflammation and apoptosis [102]. RR exhibits a protective effect against cardiac ischemia-reperfusion injury by inhibiting apoptosis through promoting mitophagy *via* inhibition of USP33 [103]. Activation of G protein-coupled estrogen receptor 1 at the onset of reperfusion protects the myocardium from I/R injury by decreasing mitophagy through the PINK1/Parkin pathway [104]. Similarly, PPENK reduces myocardial I/R injury by promoting mitophagy through the PINK1-Parkin pathway [105].

Drp1-induced disruption of mitophagy confers tolerance to lethal hypoxia-induced cellular damage [39]. Numerous studies underscore the importance of mitophagy in promoting the survival of remaining cardiomyocytes during pre-ischemic stress in ischemic cardiomyopathy. For instance, upregulation of RIPK3 prevents PINK1-Parkin-induced mitophagy by inhibiting AMPK, thus promoting necroptotic apoptosis and functional remodeling of cardiomyocytes [106]. Conversely, knocking down RIPK3 avoids necroptotic apoptosis and mitigates hypoxia-induced myocyte injury [107]. Given that myocardial infarction is accompanied by metabolic stress and AMPK activation, it can be inferred that AMPK activation leads to PINK1-Parkin mitophagy, suggesting that mitophagy decelerates the progression of necrotic death in cardiomyocytes [108]_._

Similar to the aforementioned studies, additional research has focused on the AMPK signaling pathway. Melatonin promotes cardiomyocyte survival and attenuates myocardial ischemia-reperfusion injury by enhancing mitophagy and activating the AMPK-OPA1 signaling pathway [109]. Knockdown of SIRT6 inhibits melatonin-induced AMPK-PGC-1-AKT activation, inhibiting its cardioprotective effects [110]. Moreover, melatonin has been shown to promote cardiomyocyte survival through the Apelin/SIRT3 signaling axis, inhibiting excessive mitophagy and preventing myocardial ischemia-reperfusion injury [111]. Therapeutic hypothermia-induced infarction significantly increased myocardial mitophagy, reducing cellular stress, apoptosis, inflammation, and fibrosis. These findings suggest that mitophagy is a key mechanism for cardiomyocyte recovery after myocardial infarction under hypothermia, indicating that new therapeutic strategies targeting these pathways could improve heart failure prevention [112].

Additionally, ischemia-reperfusion triggers upregulation of RIPK3, promoting phosphorylation of FUNDC1 and inducing apoptosis through FUNDC1-mediated mitophagy dysfunction [113]. Consistent findings have shown that knockdown of RIPK3 inhibits mitochondrial apoptosis and reduces the number of ischemic cardiomyocyte deaths [114].

### Mitophagy promotes inflammation

In a neonatal cardiomyocyte model of ischemia-like disease, mitophagy has been identified as an activator of nucleotide-binding oligomerization domain-like receptor protein 3 (NLRP3) inflammasomes [115]. Mechanistically, Drp1-associated mitochondrial fission facilitates the release of mtDNA and mitochondrial ROS (mtROS) into the cytoplasm, leading to the formation of inflammasomes within the myocardium and elevated transcription of pro-inflammatory factors, such as cysteine-3 and IL-18 [116].

Beyond cardiomyocytes, endothelial inflammatory responses are also influenced by mitophagy. Under hypoxic conditions, aberrant mitophagy promotes endothelial cell membrane damage and enhances monocyte-endothelial cell adhesion [117]. Given mitophagy’s essential role in inhibiting mtDNA release and mtROS leakage, its anti-inflammatory properties have been observed in neurological disorders, though this concept remains unvalidated in ischemic cardiomyopathy (ICM). Similarly, the inhibitory effects of PGC1 or Nrf-2-dependent mitochondrial biogenesis have been studied in diabetic cardiomyopathy [118].

Hydrogen has been confirmed as an effective therapeutic agent in many diseases associated with ischemic cardiomyopathy due to its anti-inflammatory, anti-apoptotic, and antioxidant properties. Recent studies demonstrate that hydrogen exerts these effects in ischemic cardiomyopathy-induced cardiomyocytes *via* PINK1/Parkin-mediated mitophagy [119]. Additionally, CCCP activates the PINK1/Parkin pathway and counteracts the regulation of mitophagy and mitochondrial function influenced by sanguinarine link (SL) extracts. Shenlian extract may protect the microvascular system by modulating mitophagy to inhibit inflammation progression, thereby alleviating coronary no-reflow [119]. However, further experiments are necessary to elucidate the mechanisms by which mitophagy modulates cardiac function in ischemic cardiomyopathy and influences inflammation progression.

## Clinical applications

Comprehensive proteomic analysis has revealed significant differences in cytoskeletal regulation and mitophagy between ischemic cardiomyopathy and other cardiomyopathies, such as dilated cardiomyopathy [120]. The current mechanisms of mitophagy in ischemic cardiomyopathy have been thoroughly detailed. However, with the increasing emphasis on translational medicine, it is essential for basic research to evolve towards broader clinical applications. Therefore, this review provides a detailed summary of the current progress in clinical applications in this field.

### Melatonin

Melatonin has been shown to slow the progression of cardiomyopathy by targeting mitochondrial quality control, with the activation of melatonin membrane receptors playing a critical role in cardioprotection. Melatonin inhibits mitophagy, reduces apoptosis and oxidative stress, and restores mitochondrial function [121]. Studies have demonstrated that long-term melatonin therapy attenuates the progression of ischemic cardiomyopathy and reduces myocardial vulnerability to ischemia-reperfusion injury by maintaining mitochondrial quality control. Knockdown of SIRT6 inhibits melatonin-induced activation of the AMPK-PGC-1-AKT pathway, diminishing its cardioprotective effects [114]. Similarly, melatonin prevents myocardial ischemia-reperfusion injury by inhibiting excessive mitophagy through the Apelin/SIRT3 signaling axis [113]. Additionally, melatonin has been shown to ameliorate mitophagy and activate the AMPK-OPA1 signaling pathway, promoting cardiomyocyte survival and reducing myocardial I/R injury [115]. It also protects the cardiac microvascular system from reperfusion injury through VDAC1-HK2-mPTP-induced mitophagy [122]. These studies reveal that melatonin significantly impacts the progression of ischemic cardiomyopathy through mitophagy. However, the specific aspects of mitophagy influenced by melatonin in ischemic cardiomyopathy require further exploration. Melatonin inhibits platelet activation and mitigates myocardial I/R injury *via* the PPARγ/FUNDC1 pathway [123]. Interestingly, melatonin-induced cardioprotection is not associated with the reversal of mitochondrial dysfunction or changes in the PINK1/Parkin pathway [84], suggesting that melatonin’s modulation of mitophagy may favor the FUNDC1 pathway.

### Traditional Chinese medicine (TCM)

In TCM patients, significant changes in PINK1 protein expression related to mitophagy were identified, highlighting the potential of TCM treatments for ischemic cardiomyopathy [124].

Myocardial I/R injury is the primary cause of post-ischemic heart failure exacerbation. Oxidative stress-induced mitochondrial dysfunction is crucial in the pathological progression of myocardial I/R. A Chinese herbal formulation, Yi Qi Huo Xue (YQHX), was developed and shown to alleviate I/R injury. Network analysis combined with ultra-high-performance liquid chromatography-high-resolution mass spectrometry elucidated the active components of YQHX and revealed its mitophagy-regulating mechanism in treating I/R injury [59]. In vivo experiments confirmed that YQHX significantly mitigated I/R myocardial injury and alleviated oxidative stress. In vitro experiments verified that YQHX could alleviate hypoxia/reoxygenation injury and attenuate oxidative stress by improving mitochondrial structure and function, closely related to mitophagy regulation. In conclusion, YQHX can alleviate hypoxia-induced injury and reduce oxidative stress by targeting mitophagy [59].

Quercetin can lower blood pressure, enhance capillary resistance, reduce capillary fragility, lower blood lipids, dilate coronary arteries, and increase coronary blood flow. It also has an adjunctive therapeutic effect on coronary heart disease and hypertension. Quercetin has shown regulatory effects on mitochondrial quality control and myocardial/vasculoprotective properties, making it a promising candidate for cardiovascular disease treatment. Quercetin attenuated mitochondrial oxidative stress-induced cardiomyocyte injury *via* the DNA-PKcs-SIRT5 pathway and inhibited necrotic apoptosis in cardiomyocytes after ischemia-reperfusion [81].

Previous reports have shown that RR has a protective effect against cardiac ischemia-reperfusion injury. A recent study explored RR’s effect on postresuscitation myocardial dysfunction (PRMD) and analyzed the underlying mechanisms [107]. The study established an experimental cardiac arrest model by inducing ventricular fibrillation followed by cardiopulmonary resuscitation. Intraperitoneal administration of RR at the onset of autonomic circulation recovery improved myocardial function and reduced oxidative stress markers such as malondialdehyde and ROS. RR also helped maintain mitochondrial structure, increased ATP and GTP levels, and attenuated cardiomyocyte apoptosis [107]. Upregulation of proteins closely related to mitophagy was observed, suggesting that RR reduces myocardial injury by promoting mitophagy through USP33 inhibition. These effects indicate RR’s potential as a therapeutic approach for addressing postresuscitation myocardial dysfunction [107].

Sacred link (SL) extracts have demonstrated efficacy in preventing and treating atherosclerosis and myocardial ischemia. However, the functional and molecular mechanisms of SL on coronary no-reflow injury have not been fully elucidated. In the model group, larger morphology and autophagosomes were frequently observed, whereas SL inhibited the hyperactivation of mitophagy regulated by the PINK/Parkin pathway. The results also showed that mitochondrial dysfunction stimulated endothelial cell barrier dysfunction, but SL treatment significantly reduced Evans blue transmission and increased transmembrane resistance [101]. Carbocyanine-3-chlorophenylhydrazone (CCCP) activated the PINK/Parkin pathway and reversed the regulation of mitophagy and mitochondrial function by SL. In conclusion, Shenlian extract blocked the activation of the PINK/Parkin pathway and inhibited excessive mitophagy to modulate mitochondrial dysfunction, thereby inhibiting myocardial ischemia-reperfusion injury [101].

Other studies have confirmed that concentrao alleviates myocardial ischemia-reperfusion injury primarily through the activation of Parkin-mediated mitophagy and the down-regulation of the ubiquitin-proteasome system [125]. However, this study only provided a superficial observation of changes in mitophagy indexes and the effects on ischemic cardiomyopathy at different doses of concentrao, without an in-depth exploration of these effects.

In conclusion, these studies collectively confirm that traditional Chinese medicine is playing a pivotal role in the field of mitophagy and its impact on ischemic cardiomyopathy.

### Other therapeutic measures

Although some clinical drugs have not gained widespread use in the field of mitophagy affecting ischemic cardiomyopathy compared to traditional Chinese medicine, notable research progress has been made. Studies have confirmed that dexprazoxane attenuates myocardial I/R injury through the upregulation of mitophagy *via* PINK1 and Parkin [126]. Additionally, donepezil administration has been found to reduce myocardial I/R injury by decreasing apoptosis and oxidative stress while increasing mitophagy [127]. Similarly, dexmedetomidine pretreatment attenuates myocardial I/R injury by activating the α2-adrenergic receptor, which inhibits mitophagy and reduces injury [128] Thyroid hormone treatment also provides cardioprotection by enhancing PINK1-dependent mitophagy, reducing cell death, and preventing I/R injury [105]. Combining TCM with clinical medications has shown promising results. For instance, combined treatment with metformin (MET) and ginseng dingzhi tang (GN) in hyperglycemic cardiomyocytes can indirectly increase mitophagy to attenuate myocardial injury [129].

Beyond clinical drugs, novel therapies are emerging. Exercise has been shown to attenuate redox signaling-induced acetylcholine receptor effects in myocardial ischemia-reperfusion injury by modulating mitophagy [130]. Therapeutic hypothermia-induced infarction significantly increases myocardial mitophagy, mitochondrial mass, and function, leading to reductions in cellular stress, apoptosis, inflammation, and fibrosis [131]. These findings suggest that mitophagy is essential for cardiomyocyte recovery post-myocardial infarction under hypothermia. Furthermore, electroacupuncture preconditioning attenuates myocardial ischemia-reperfusion injury by inhibiting mitophagy mediated by the mTORC1-ULK1-FUNDC1 pathway [116]. Targeting these pathways with novel therapeutic strategies may enhance heart failure prevention (Table 1).

**Table 1:** Compounds or drugs targeting mitophagy in ischemic cardiomyopathy.

Drug name	Mechanism of action	Reference
melatonin	Knocking down SIRT6 can inhibit melatonin induced AMPK-PGC-1-AKT activation and its cardioprotective effect	[103]
melatonin	Melatonin has been found to inhibit excessive mitophagy through the Apelin/SIRT3 signaling axis, preventing myocardial ischemia-reperfusion injury	[104]
melatonin	Melatonin can promote myocardial cell survival and alleviate myocardial ischemia-reperfusion injury by improving mitophagy and activating the AMPK-OPA1 optic atrophy 1 (OPA1) signaling pathway	[102]
melatonin	Melatonin protects the cardiac microvascular system from reperfusion injury through VDAC1-HK2-mPTP induced mitophagy	[111]
melatonin	Melatonin can inhibit platelet activation and protect against myocardial ischemia/reperfusion injury through the PPAR /FUNDC1 pathway	[112]
YQHX	YQHX can alleviate hypoxia/reoxygenation damage and reduce oxidative stress by improving the structure and function of mitochondria, which is closely related to regulating mitophagy.	[68]
Quercetin	Quercetin alleviates myocardial cell damage caused by mitochondrial oxidative stress and inhibits necrotic apoptosis of myocardial cells after ischemia-reperfusion through DNA-PKcs-SIRT5	[70]
RR	RR can weaken PRMD by inhibiting USP33 and promoting mitophagy	[96]
Shenlian	The use of Shenlian extract prevented the activation of the PINK/Parkin pathway and inhibited excessive mitophagy to regulate mitochondrial dysfunction and suppress myocardial ischemia-reperfusion injury	[90]
Tongxin Luo	Tongxin Luo mainly improves myocardial ischemia-reperfusion injury by activating Parkin mediated mitophagy and downregulating the ubiquitin protease system	[114]
Dexmedetomidine	Dexmedetomidine upregulates mitophagy through PINK1 and Parkin to alleviate myocardial ischemia/reperfusion injury	[115]
Donepezil	Administration of donepezil alleviates myocardial ischemia/reperfusion injury by reducing cell apoptosis, oxidative stress, and increasing mitophagy	[116]
Dexmedetomidine	Pre treatment with dexmedetomidine inhibits mitophagy by activating 2-adrenergic receptors, reducing myocardial ischemia/reperfusion injury	[117]
Thyroid hormone	Thyroid hormone treatment has also been shown to provide cardiac protection, reduce cell death, and prevent I/R injury by enhancing PINK1 dependent mitophagy	[94]
MET + GN	Further intervention in high glucose cardiomyocytes through the combination therapy of metformin (MET) and ginseng dingzhi decoction (GN) can indirectly enhance mitophagy to alleviate myocardial injury	[119]

## Discussion

In recent years, the role of the mitochondrial control system in ischemic heart disease has become a primary focus of research. Mitophagy, a key component of this system, plays a unique role in ischemic heart disease, distinct from mitochondrial dynamics. Most systematic reviews have broadly grouped mitophagy with the mitochondrial control system without delving into its specific functions in ischemic heart disease. This paper aims to summarize the progress in understanding how mitophagy regulates ischemic heart disease.

**Fig. 4:**
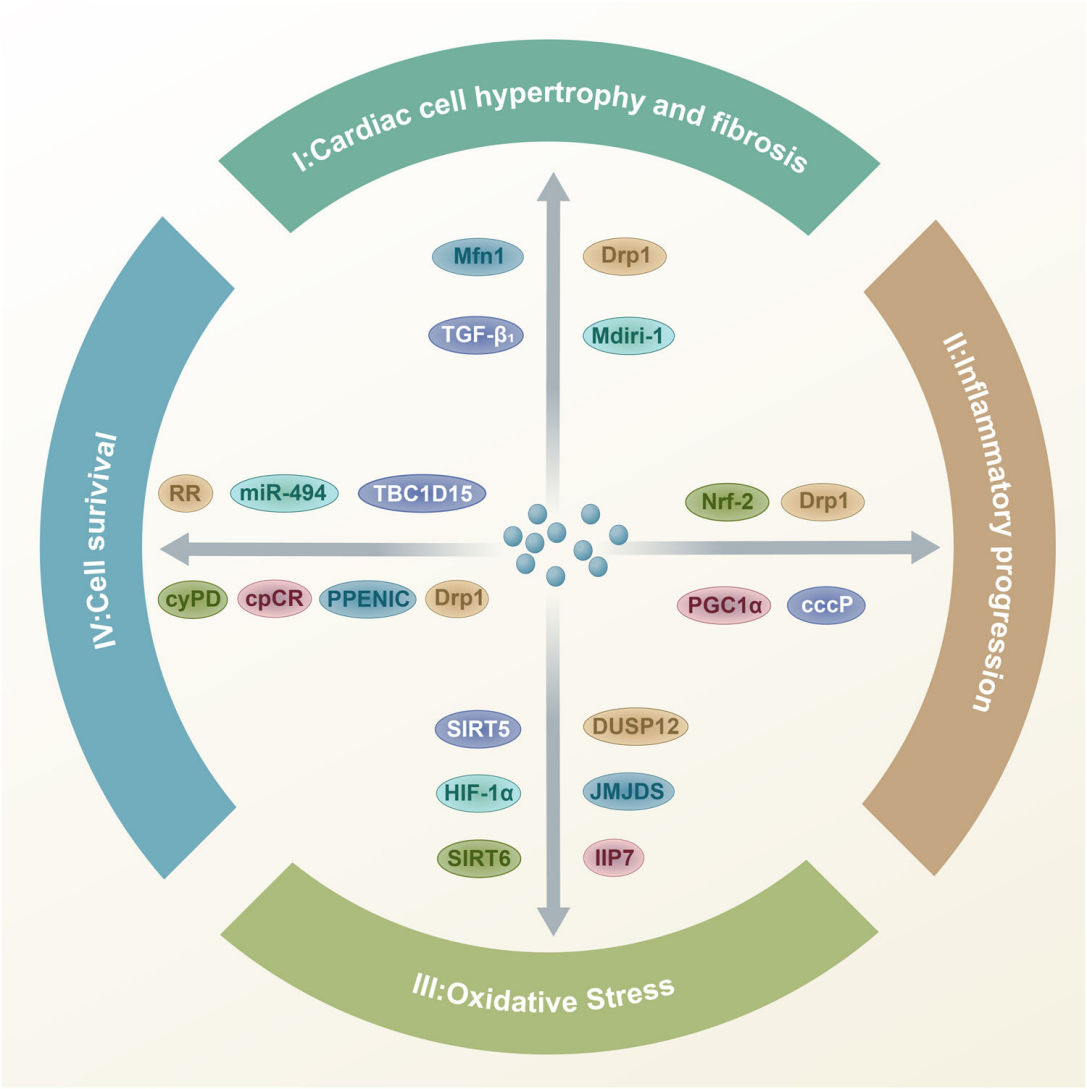
Summary of the role of mitophagy in promoting the progression of ischemic heart disease: 1. Mitophagy promotes myocardial cell hypertrophy and fibrosis. 2. Mitophagy promotes inflammation. 3. Mitophagy promotes oxidative stress progression. 4. Mitophagy promotes cell death.

By analyzing recent relevant articles, this study identifies four main ways mitophagy regulates ischemic heart disease: promoting cardiomyocyte hypertrophy and fibrosis, advancing oxidative stress, facilitating inflammation, and inducing cardiomyocyte death **(**Fig. 4).

Myocardial fibrosis, a pathological change in cardiac structure due to various causes of myocardial injury, is influenced by mitophagy. Knockdown of genes such as Drp1, Mfn1, or Mfn2 in cardiomyocytes inhibits mitochondrial fission, leading to mitochondrial enlargement. These abnormal mitochondria inhibit mitophagy, affecting cardiomyocyte hypertrophy and causing damage. Similarly, mitophagy partially contributes to myocardial fibrosis. Interventions targeting TGF-β1 and Mdivi-1 inhibit mitophagy, alleviating fibrosis in the border zone of myocardial infarction, indicating that mitophagy promotes cardiomyocyte hypertrophy and fibrosis.

Ischemia and hypoxia disrupt the mitochondrial quality control system, leading to mitochondrial dysfunction and a surge in ROS. Studies have shown that melatonin can upregulate mitophagy and reverse cellular damage caused by myocardial ischemia. Mitochondria, acting as secondary calcium reservoirs in cardiomyocytes, buffer Ca^2+^ fluctuations. Excessive mitochondrial calcium accumulation causes dysfunction, leading to cardiomyocyte damage. Increased mitochondrial fission triggers oxidative stress in ischemic cardiomyocytes, affecting mitophagy and subsequently regulating cardiomyocyte health. Zn^2+^, like Ca^2+^, plays a significant role in regulating mitochondrial oxidative stress, highlighting the importance of iron metabolism in mitophagy-mediated myocardial ischemia. In conclusion, mitophagy critically influences the progression of ischemic heart disease by impacting oxidative stress, and this should be a central focus of research in this field.

Drp1-associated mitochondrial fission has been shown to promote the formation of inflammatory vesicles and pro-inflammatory factors (e.g., cysteine-3 and IL-18), accelerating the progression of ischemic heart disease. Additionally, CCCP has demonstrated protective effects on the microvascular system by activating the PINK/Parkin pathway to inhibit inflammation, thereby alleviating coronary no-reflow. However, further experiments are necessary to elucidate the mechanisms through which cardiac mitophagy regulates ischemic cardiomyopathy by modulating inflammation.

Numerous studies have emphasized the critical role of mitophagy in promoting the survival of cardiomyocytes during pre-ischemic stress in ischemic cardiomyopathy. Mitophagy influences signaling pathways such as AKT, AMPK, and SIRT3, affecting PINK1-Parkin and other pathways, thereby promoting cardiomyocyte apoptosis. In conclusion, mitophagy plays a pivotal role in cardiomyocyte hypertrophy and fibrosis, the progression of oxidative stress, inflammation, and cardiomyocyte death.

Recent research in this area has yielded significant clinical translational results. Many drugs have been found to protect the myocardium by modulating mitophagy. Notably, traditional Chinese medicine has shown promising results in this field. However, the emergence of drug resistance necessitates the development of new treatments. Compared with traditional Western medicines, Chinese medicines effectively reduce renal toxicity. Future research should focus on combining Chinese and Western medicines for ischemic cardiomyopathy treatment, as this combination may enhance cardiomyocyte protection.

However, it is undeniable that this field, although some progress has been made so far, still has some problems to which we must pay attention.

First, most of the current studies on mitophagy affecting ischemic cardiomyopathy are still in the exploratory stage, and most of them have not penetrated to the genetic level as the basic studies in other fields have already done. According to the current mainstream conclusion, it should be accurate that mitophagy can influence the progression of ischemic cardiomyopathy, but through which pathway does mitophagy occur? Which pathways are the key signaling pathways involved? Is the role of mitophagy in ischemic cardiomyopathy specific to other types of cardiomyopathy? These are all factors that we need to consider.

Second, many studies have attempted to reveal the role of mitophagy in ischemic cardiomyopathy, but we need to consider whether it is the right decision to limit ourselves too much to mitophagy. Whether it is other types of autophagy besides mitophagy, or other factors in mitochondrial dynamics besides mitophagy, do they also have an impact on ischemic cardiomyopathy? Does mitophagy play a critical and decisive role in the development of ischemic cardiomyopathy? All these need to be confirmed by subsequent studies. At present, with the continuous updating of single-cell sequencing and various advanced means of big data analysis, the analysis of a large number of samples, coupled with relevant experimental verification, may be able to confirm the special significance of mitophagy in ischemic cardiomyopathy. Of course, we can not only stay in the perspective of mitophagy, we also need to consider a variety of factors, after all, the microenvironment of cardiomyocyte survival is composed of a variety of different substances, for example, endothelial cells and blood cells including platelets may also play a role in mitophagy affecting cardiomyocytes. Therefore, these factors, which may have been overlooked before, may be a high-potential area for future research.

In addition to the above questions we need to think about, we must also focus on mitophagy itself. mitophagy, as a type of autophagy, also possesses the classic ‘double-edged sword’ effect of autophagy. Based on our results above, we tentatively conclude that mitophagy can promote cardiomyocyte hypertrophy and fibrosis, exacerbate oxidative stress, promote inflammation, and induce cardiomyocyte death. However, does mitophagy exert only negative effects in cardiomyocytes? Does mitophagy protect cardiomyocytes from stressful changes in the microenvironment? This is something that needs to be explored in subsequent research goes.

In addition, it must be clear that most of the results obtained so far on the effects of mitophagy on ischemic cardiomyopathy are specific and not sufficient to be generalized to all patients with ischemic heart disease. This is because the role played by mitophagy may be due to different factors: different stress conditions, the patient’s age, genetic disorders, and the presence or absence of other concomitant organic diseases, which are all worth considering.

In any case, mitophagy and the mitochondrial control system are inextricably linked, and future research should also be directed toward exploring the specific mechanisms underlying the dual roles of mitophagy and mitochondrial dynamics in ischemic heart disease.

In conclusion, we have elaborated the specific mechanisms by which mitophagy currently regulates ischemic cardiomyopathy, aiming to be better able to provide more means of protecting cardiomyocytes in the future. We believe that this field will definitely become a hotspot of research in ischemic heart disease in the future.
